# Antiviral effect of compounds derived from the seeds of *Mammea americana* and *Tabernaemontana cymosa* on Dengue and Chikungunya virus infections

**DOI:** 10.1186/s12906-017-1562-1

**Published:** 2017-01-18

**Authors:** Cecilia Gómez-Calderón, Carol Mesa-Castro, Sara Robledo, Sergio Gómez, Santiago Bolivar-Avila, Fredyc Diaz-Castillo, Marlen Martínez-Gutierrez

**Affiliations:** 1 Grupo de Investigación en Ciencias Animales-GRICA, Universidad Cooperativa de Colombia, Calle 30A # 33-51, Bucaramanga, Colombia; 2 Facultad de Ciencias de la Salud, Programa de Bacteriología y Laboratorio Clínico, Grupo de investigación en manejo clínico - CLINIUDES, Universidad de Santander UDES, Bucaramanga, Colombia; 3 Maestría en Enfermedades Infecciosas, Universidad de Santander, Bucaramanga, Colombia; 4 PECET, Facultad de Medicina, University of Antioquia, Medellín, Colombia; 5 Laboratorio de Investigaciones Fotoquímicas y Farmacológicas de la Universidad de Cartagena (LIFFUC), Universidad de Cartagena, Cartagena, Colombia

**Keywords:** Dengue Virus, Chikungunya Virus, Antiviral, *Mammea americana*, *Tabernaemontana cymosa*

## Abstract

**Background:**

The transmission of Dengue virus (DENV) and Chikungunya virus (CHIKV) has increased worldwide, due in part to the lack of a specific antiviral treatment. For this reason, the search for compounds with antiviral potential, either as licensed drugs or in natural products, is a research priority. The objective of this study was to identify some of the compounds that are present in *Mammea americana* (*M. americana*) and *Tabernaemontana cymosa (T. cymosa)* plants and, subsequently, to evaluate their cytotoxicity in VERO cells and their potential antiviral effects on DENV and CHIKV infections in those same cells.

**Methods:**

Dry ethanolic extracts of *M. americana* and *T. cymosa* seeds were subjected to open column chromatographic fractionation, leading to the identification of four compounds: two coumarins, derived from *M. americana;* and lupeol acetate and voacangine derived from *T. cymosa.*. The cytotoxicity of each compound was subsequently assessed by the MTT method (at concentrations from 400 to 6.25 μg/mL). Pre- and post-treatment antiviral assays were performed at non-toxic concentrations; the resulting DENV inhibition was evaluated by Real-Time PCR, and the CHIKV inhibition was tested by the plating method. The results were analyzed by means of statistical analysis.

**Results:**

The compounds showed low toxicity at concentrations ≤ 200 μg/mL. The compounds coumarin A and coumarin B, which are derived from the *M. americana* plant, significantly inhibited infection with both viruses during the implementation of the two experimental strategies employed here (post-treatment with inhibition percentages greater than 50%, *p* < 0.01; and pre-treatment with percentages of inhibition greater than 40%, *p* < 0.01). However, the lupeol acetate and voacangine compounds, which were derived from the *T. cymosa* plant, only significantly inhibited the DENV infection during the post-treatment strategy (at inhibition percentages greater than 70%, *p* < 0.01).

**Conclusion:**

In vitro, the coumarins are capable of inhibiting infection by DENV and CHIKV (with inhibition percentages above 50% in different experimental strategies), which could indicate that these two compounds are potential antivirals for treating Dengue and Chikungunya fever. Additionally, lupeol acetate and voacangine efficiently inhibit infection with DENV, also turning them into promising antivirals for Dengue fever.

## Background

Arboviruses are viruses that are transmitted from one vertebrate host to another by hematophagous mosquitoes, the majority of them belonging to the *Diptera* order [1, 2]. These viruses are grouped primarily into the families *Flaviviridae*, *Togaviridae*, and *Bunyaviridae*, and they are capable of producing diseases in both humans as well as in animals [3]. Viruses of the genus *Flavivirus* are found within the *Flaviviridae* family, in which the most important representative is the Dengue virus (DENV) [4], and the *Alphavirus* genus is within the *Togaviridae*se family, with the Chikungunya virus (CHIKV) being its most important representative [5]. These two viruses are transmitted by mosquitoes of the genus *Aedes*, with *Aedes aegypti* being the most proficient vector because it has largely been urbanized [6].

DENV is an enveloped virus with icosahedral symmetry, a single-stranded RNA genome and positive polarity (approximate length, 10.7 Kb) [7]. The genome encodes a single viral polyprotein that gives rise to three structural proteins designated as the Capsid (C), Pre-Matrix/Matrix (prM/M), and Envelope (E) and to seven nonstructural proteins designated NS1, NS2A, NS2B, NS3, NS4A, NS4B, and NS5 [8]. Each of these proteins fulfills important functions during entry and viral replication in the host cell. The DENV infection process is initiated through the binding of the virus to receptors on the cell surface via the E protein, followed by endocytosis, in which variations in pH trigger the fusion of this protein with the endosomal membrane, releasing the nucleocapsid (RNA bound to the C protein) into the cytoplasm. After this release, the transcription process (which generates negative intermediary RNAs and subsequently new positive sense RNA) and translation start in ribosomes associated with the endoplasmic reticulum. The newly generated polyprotein is cleaved by cellular and viral proteases for assembly with the viral RNAs, with new viruses being released by gemmation [9].

CHIKV is an enveloped virus with spherical symmetry, a single-stranded RNA genome and positive polarity (approximate length, 12 Kb) [10]. Its genome encodes a structural polyprotein and a nonstructural protein, which are in turn cleaved into five structural proteins (C, E3, E2, 6 K, and E1) and four nonstructural proteins (NSP1, NSP2, NSP3, and NSP4), respectively [11]. CHIKV begins its infection process by binding the receptors that are present on the cell membrane with the E1 and E2 proteins. Clathrin-mediated endocytosis subsequently occurs, which leads to the denudation of the viral genome. After the translation of the RNA, the nonstructural viral proteins (which are responsible for replicating the viral genome) and structural proteins are produced, which enable the assembly of new viral particles that are released by gemmation [12].

Globally, Dengue is the most important arbovirus, with epidemics reported in more than 100 countries in Asia, Africa, and the Americas [13]. Although Chikungunya infects smaller percentages than Dengue, it has also impacted global health, jeopardizing the same geographical regions in which Dengue is present [14]. In the Americas, both Dengue and Chikungunya are reasons for frequent medical consultation, but a specific treatment does not exist for either case. Control strategies for these diseases are focused on three different fronts [15]. The first of these approaches involves vector control through community education, the elimination of breeding sites, fumigation, and biological intervention, among others [16]. However, these strategies have not been totally effective, as evidenced by the re-emergence of the mosquito vector and, hence, of the disease in areas where it had already been eradicated [17]. The next front is the implementation of vaccines. In the case of Dengue, several vaccines are in phases II and III of development [18], including in some countries in Latin America and Asia. The first vaccine (CYD-TDV-Dengvaxia Sanofi Pasteur) was licensed for use in the population between 9 and 45 years of age living in endemic areas [19]. However, it is important to account for the effectiveness of the vaccine, the level of protection and, therefore, its usefulness. The usefulness could be affected by the immune response that the vaccine induces against the four serotypes because if the protection is not adequate, the antibody-dependent enhancement phenomenon could be triggered, favoring the development of the disease [20]. However, there is little research on vaccines for preventing these diseases, and none are licensed at this time [21]. Finally, the third front includes the search for cost-effective, low-toxicity antiviral drugs (either secondary use medications or components of natural products) that achieve a prophylactic and/or therapeutic effect. Many advances have been achieved with this scenario, for both Dengue [22] as well as for Chikungunya [23], but at this time, there is no licensed drug that can be used in the infected population.

For centuries, plants have acted as sources of compounds with biological properties, among which are included antiviral effects against viruses such as DENV [24] and CHIKV [25], demonstrating their ability to inhibit some of the viral replication cycle processes in the cell (from entry to the release of new viruses). In this context, our working group recently showed that extracts obtained from plants in the Colombian Caribbean region significantly inhibit DENV infection in cell culture [26]. Among these plants are *Mammea americana* (*M. americana*) and *Tabernaemontana cymosa* (*T. cymosa*).

*M. americana* (Family *Clusiaceae*) is native to the Caribbean and Central America and is known as a fruit tree that is distributed throughout tropical and temperate regions [27]. *T. cymosa* (Family *Apocynaceae*) is originally from Colombia, Venezuela, and Trinidad, and it is also distributed throughout the tropical and subtropical regions of the world [28]. The biological activities of these plants, such as antimicrobial, antiparasitic, antitumoral, antifebrile, analgesic, and antiviral properties, have been widely studied [29, 30], as well as their effects against the larvae and adults of *A. aegypti* [31].

Taking this background into account, the objective of this study was to identify some of the compounds that are present in the *M. americana* and *T. cymosa* plants and to subsequently evaluate their cytotoxicity in VERO cells and their potential antiviral effect on DENV and CHIKV infection in those same cells.

## Methods

### Obtaining extracts from plant material

Plant selection was based on the results of an ethnobotanical survey conducted in the city of Cartagena (Colombia) and on previous reports of antiviral activity of these plants against other viruses causing febrile symptoms. The plants were collected in the Colombian Caribean Region and different parts of each plant were identified in the herbarium of the Botanical Garden Guillermo Piñeres (Cartagena, Colombia): *M. americana* Vocucher No. JBC 467 and *T. cymosa* Voucher No. JBC 3243. The plant material was macerated with 90% ethanol overnight, and the resulting extract was filtered and concentrated in a rotary evaporator. The dry ethanolic extract was resuspended in an ethanol 0.1% distilled water solution and stored at −70 °C until further use.

### Chromatographic fractionation of the ethanol extracts

12.4 g sample of dry total ethanolic extract from *M. americana* (FD-I-34S) seeds was absorbed onto 12 g of silica gel and dried at room temperature. The extract was subsequently subjected to normal-phase open-column chromatographic fractionation using silica gel (Merck®, 70–230 mesh, 120 g) as the stationary phase and suspended in chloroform as the initial mobile phase. The column was eluted by employing gradients of increasing polarity, starting with chloroform and ending with methanol. Furthermore, 15 g of dry total ethanolic extract from *T. cymosa* (FD-I-26S) seeds was subjected to open-column chromatographic separation (5 cm × 60 cm), using silica gel (Merck®, 70–230 mesh, 200 g) as the stationary phase, which was suspended in dichloromethane. The ethanolic extract of the seeds was eluted using gradients of increasing polarity, starting with dichloromethane (CH_2_Cl_2_), followed by 7:3 dichloromethane/acetone, 1:1 acetone/methanol, and methanol (MeOH).

### Maintenance of viruses and cells

VERO epithelial cells (*Cercopithecus aethiops*) were acquired from the American Type Cell Collection (ATCC® CRL-1587™), and C6/36HT cells from *A. albopictus* mosquito larvae were donated by Dr. Guadalupe Guzmán from the Department of Virology at the Instituto Pedro Kouri [Pedro Kouri Institute] (Havana, Cuba). The VERO cells were maintained in Dulbecco’s modified Eagle medium (DMEM) supplemented with 2% Fetal Bovine Serum (FBS) and incubated at 37 °C in a 5% CO_2_ atmosphere. The C6/36HT cells were maintained at 34 °C in DMEM supplemented at 10%. The DENV-2/NG strain was donated by Dr. Jorge Osorio of the Department of Pathobiological Sciences, University of Wisconsin (Madison, WI, USA). Antiviral assays for CHIKV were performed by Colombian clinical isolation (PECET, Universidad de Antioquia [University of Antioquia]), and they belonged to the Asian lineage (CHIKV-ACol).

### Determination of the cytotoxic activity

Cytotoxicity was determined by the MTT (3-(4,5-dimethylthiazol-2-yl)-2,5-diphenyltetrazolium bromide) method. For this purpose, 2.5 × 10^4^ VERO cells were seeded into 96-well plates for 24 h. Then, serial dilutions of the compounds were performed (at concentrations from 200 μg/mL to 0.8 μg/mL), which were added to the cells after infection with DENV-2/NG (MOI: 1) or CHIKV-ACol (MOI: 0.1), and they were left in contact with the cells for 48 h. After this incubation period, an MTT solution (0.5 mg/mL) was added to the cultures and incubated for an additional 3 h at 37 °C. Finally, dimethyl sulfoxide was added, and the absorbance was read at 450 nm in a microplate reader Benchmark® (BioRad) Spectrophotometer. Cultures without the compounds were used as positive controls for viability. The CC_50_ (50% cytotoxicity concentration) was calculated as the extract concentration that reduced the cell viability by 50% by means of regression analysis. Each experimental condition was evaluated in triplicate over two independent experiments (n: 6).

### Determining the antiviral effect of the fractions and compounds on viral entry into cells

For this purpose, 2.5 × 10^4^ VERO cells were seeded into 96-well plates for 24 h, and then the compounds (at a concentration of 200 μg/mL) were added and left in contact with the cells for 48 h according to the previously described methodology [32, 33]. Subsequently, the treatment was removed, and the viral inoculum (the DENV-2/NG strain at an MOI of 1 or the CHIKV-ACol strain at an MOI of 0.1) was added and left for 2 h. It was then removed, and fresh medium was added for an additional 48 h. Once the time was up, the supernatants were harvested and stored at −70 °C until they were processed by the titration technique for plating (to quantify CHIKV) or by real-time RT-PCR (to quantify DENV). In each case, two independent experiments, each with two replicates, were performed (n: 4).

### Determining the antiviral effect of the fractions and compounds on the steps subsequent to viral entry into the cells

For this phase, 2.5 × 10^4^ VERO cells were seeded into 96-well plates for 24 h, and then the viral inoculum (the DENV-2/NG strain at an MOI of 1 or the CHIKV-ACol strain at an MOI of 0.1) was added for 2 h. The inoculum was then removed, and the compounds (concentrations from 200 μg/mL to 0.8 μg/mL) were added and left in contact with the cells for an additional 48 h [32, 33]. Once the time was completed, the supernatants were harvested and stored at −70 °C until they were processed by the titration technique for plating (to quantify CHIKV) or by real-time RT-PCR (to quantify DENV). In each case, two independent experiments, each with two replicates, were performed (n: 4).

### Quantification of CHIKV

The infectious viral particles of CHIKV were quantified by microtitration technique for plating. In summary, 2.5 × 10^4^ VERO cells were seeded into 96-well plates for 24 h. Then, serial dilutions of the harvested supernatants were performed (1 × 10^−1^ to 1 × 10^−5^) and inoculated onto the cells for 2 h. The supernatants were subsequently removed, and the cells were incubated with 1.5% carboxymethylcellulose (Sigma-Aldrich, St. Louis, MO) prepared in DMEM that had been supplemented with 2% FBS for 72 h at 37 °C in a CO_2_ atm. After that time, the monolayers were fixed with 4% paraformaldehyde (Sigma-Aldrich) and stained with 1% crystal violet (Sigma-Aldrich). The plates were counted to determine the number of plaque-forming units (PFU/mL). Each of the replicates was titrated in duplicate.

### Quantification of DENV

The DENV genome was quantified by real-time PCR. For this purpose, a viral RNA extraction was performed with a Qiagen® extraction kit (QIAamp® Viral RNA Mini Kit) according to the protocol described by the manufacturer. The quality and quantity of RNA was determined by spectrophotometric analysis in a NanoDrop™ 2000c UV-vis spectrophotometer (Thermo Scientific®), and the quantified RNA was stored at −70 °C until use. cDNAc synthesis was performed with a RevertAid™ First Strand cDNA Synthesis Kit (Thermo Scientific®) according to the manufacturer’s instructions, using 0.5 μg of RNA and random primers for retrotranscription. The cDNA was stored at −70 °C until use. The cDNA was subsequently amplified by real-time PCR (qPCR) using a PowerUp™ SYBR™ Green Master Mix Kit (Thermo Fisher Scientific Amplification) and the following primers, which have also been described previously: mD1-F-5’-TCA ATA TGC TGA AAC GCG AGA GAA ACC G-3’ and mTS2-F-5’-CGC CAC AAG GGC CAT GAA CAG TTT-3’. These primers amplify a 119 bp segment of the C-prM region of the viral genome. Amplification was performed in a Bio-Rad CFX96TM Real-Time System C 1000 Thermal Cycler, and the genomic copies were calculated using a specific standard curve for DENV-2 that was constructed previously [34]. The results are expressed as the mean of four independent experiments (*n* = 4).

### Data analysis

To determine the CC_50_ (50% cytotoxicity concentration) and the EC_50_ (50% effective concentration), a regression analysis was performed. The Selectivity Index (SI) of each molecule was determined from the relationship between the CC_50_ and the EC_50_, with the formula SI = CC_50_/EC_50_. To compare the cell viability between the cultures that were treated with the compounds and the untreated cultures, ANOVA was used, followed by a test of Minimum Significant Difference. To compare the number of infectious viral particles that were released (CHIKV) or the number of viral copies (DENV) between the cells that were treated with each molecule during the pre-treatment strategy and for the untreated cells, Student’s t-test was used. To compare the number of infectious viral particles that was released (CHIKV) or the number of viral copies (DENV) between the cells that was treated with each molecule during the post-treatment strategy with the untreated cells, ANOVA was used, followed by a test of Minimum Difference. All statistical analyses were performed using Prism® 7.01 software for Windows™ (GraphPad Software, San Diego, CA). All cases with a p-value of less than 0.05 (*p* < 0.05) were considered to have a statistically significant difference.

## Results

### Identification of the compounds present in the *M. americana* and *T. cymosa* fractions

**Fig. 1:**
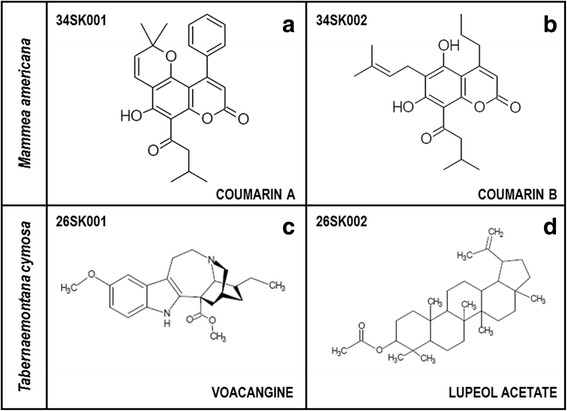
Structures of the compounds that were identified from the seeds of *M. americana* and *T. cymosa*. They were identified by using Nuclear Magnetic Resonance in one and two dimensions (1D and 2D NMR) and mass spectrometry (MS) and were compared with data reported in the literature. **a** and **b** Coumarins derived from *M. americana*. **c** Voacangine derived from *T. cymosa*. **d** Lupeol acetate derived from *T. cymosa*

Five fractions were obtained from the *M. americana* extract (FD-I-34S) and four fractions were obtained from the *T. cymosa* extract (FD-I-26S). A preliminary antiviral screening was performed to the fractions obtained from the extracts (Data not shown) to proceed with the caractherization of the more promisory fractions. In that order, the compounds 34SK001 and 34SK002 were obtained from fraction 34SF03 (from *M. americana)* and compounds 26SK001 and 26SK002 were identified from fraction 26SF01 (from *T. cymosa*). The consolidated results of the open column chromatography can be observed in Table 1. The compounds were subsequently identified by using Nuclear Magnetic Resonance in one and two dimensions (1D and 2D NMR) and mass spectrometry (MS) and by comparison with the data reported in the literature. The structures were obtained from the Orbitrap database (LTQ Orbitrap, ThermoElectron-Corporation) and can be found in Fig. 1.

**Table 1:** Normal phase open column chromatography of the total extracts from *T. cymosa seeds* and *M. americana* seeds

	Fraction Code	Weight (g)	Performance (%)	Elution solvent
*Tabernaemontana cymosa* (FD-I-26S)	26SF01	9.852	65.7	CH_2_Cl_2_
	26SF02	2.141	14.3	CH_2_Cl_2_/Acetone 7:3
	26SF03	0.015	0.1	Acetone/MeOH 1:1
	26SF04	1.134	7.6	MeOH
*Mammea americana (FD-I-34S)*	34SF01	0.042	0.3	Hexane
	34SF02	6.399	51.6	Hexane/CH_2_Cl_2_ 8:2
	34SF03	2.575	20.8	CH_2_Cl_2_
	34SF04	1.994	16.1	CH_2_Cl_2_/Acetone 1:1
	34SF05	0.91	7.3	EtOH

The compounds 34SK001 and 34SK002 were obtained from fraction 34SF03 of *M. americana*

The compounds 26SK001 and 26SK001 were obtained from fraction 26SF01 of *T. cymosa*

Compound 34SK001 exhibited the following physical and spectral properties: yellow crystals; MP: 162-164 °C; ^1^ **H NMR** (300 MHz CDCl_3_):14.84 (1H, *s*,OH-7), 7.44 (3H, *dd,*H-3’/H4’/H5’), 7.36 (2H, *dd*, 2’-H/6’-H) 6.94 (1H, *d*, *J* = 9 Hz, H-4”), 6.03 (1H, *s*,H-3), 5.68 (1H, *d*, 3”-H), 3.01 (2H, *d*, *J* = 12Hz,H-2”’), 2.27 (1H, *ddd*, *J* = 6, 12, 18 Hz,H-3”’), 1.62 (2H, *s*, H-4”’/H-5”’), 1.01 (1H,*d*, *J* = 6 Hz, H-2”) ppm. ^13^ **C NMR** (75 MHz, CDCl_3_): 206.91 (C-1”’), 164.6 (C-7) 159.82 (C-2), 158.28 (C-5), 156.56 (C8a), 154.95 (C-4), 139.9 (C-1’), 126.48 (C3”), 115.69 (C-4”), 112.85 (C-3), 107.32 (C-6), 102.36 (C-4a), 80 (C-2”), 53.76 (C2”), 53.76 (C-2”’), 28.43, 25.25 (C-3”’), 22.80 (C-5”’). This compound was identified as 5-hydroxy-2,2-dimethyl-6-(3-methylbutanoyl)-10-phenyl-2H,8H-pyrano[2,3-f]cromon-8-one, by comparing its spectral and physicochemical data with those reported in the literature (coumarin A) (Fig. 1a). Furthermore, compound 34SK002 exhibited the following physical and spectral properties: yellow crystals; MP:115–116 °C; ^1^ **H NMR** (300 MHz, CDCl_3_): 14.74 (1H, *s*, OH-7), 6.91 (1H, *s,* OH-5), 6.06 (1H, *s*,H-2), 5.27 (1H,*m*,H-2”), 3.54 (1H, *d*,*J* = 9 Hz,H-1’), 3.21 (2H, *d*, *J* = 6 Hz, H-2”’), 2.97 (2H,*d*, H-1”), 2.32 (1H, *m*, H3”’), 1.90 (1H, *d*, *J* = 15 Hz, H-4’), 1.69 (3H, *dd*, *J* = 9, 15 Hz, H-3”), 1.31 (3H, *dd*, *J* = 3, 9 Hz, H-5’), 1.07 (6H, *m*, H-4”’/H-5”’) ppm. ^**13**^ **C NMR** (75 MHz, CDCl_3_): 206.5 (C-1”’), 165.9 (C-7), 159.5 (C-2), 158.4 (C-5), 157.3 (C-4), 138.5 (C-3’), 120 (C-2’), 110.4 (C-6), 106.3 (C-3), 104.2 (C-8), 100.4 (C-4a), 53.77 (C-2”’), 37.3 (C-1”), 25.97 (C-4’), 25.6 (C-3”’), 22.79 (C-2”), 21.7 (C-1’), 14.1 (C-3”). This compound was identified as 5,7-dihydroxy-6-(3-methylbut-2-en-1-yl)-8-(3-methylbutanoyl)-4-propyl-2Hcromon-2-one by comparing its spectral and physicochemical data with those reported in the literature (coumarin B) (Fig. 1b).

Compound 26SK001 exhibited the following physical and spectral properties: crystalline needles: MP: 137–138 °C; ^1^ **H NMR** (300 MHz, CDCl_3_) 7.67(1H, *s*, N-H), 7.16 (1H, *d, J =* 8.7Hz, H-12), 6.94 (1H, *d*, *J =* 2.1Hz, H-9), 6.83 (1H, *dd*, *J* = 2.7 Hz y 11.1 Hz, H-11), 3.87 (3H, *s,* OMe), 3.73 (3H, *s*, CO_2_Me) 3.56 (1H, *s*, H-21), 3.38 (1H, *m*, H-5ß), 3.23 (1H, *m*, H-5a), 3.15 (1H, *m*, H6), 3.00 (1H, *m*, H-6), 2.93 (1H, *m*, H-3ß), 2.83 (1H, *d, J* = 8.4 Hz, H-3a), 2.59 (1H, *m*, J = H-17b), 1.90 (1H, *m*, H-14), 1.75 (1H, *m,* H-15ß), 1.60 (1H, *s*, H-19ß), 1.45 (1H, *m*, H-19a), 1.33 (1H, *m*, H-20), 1.14 (1H, *m*, H-15a), 0.91 (3H, *t*, H-18). ^**13**^ **C NMR** (75 MHz, CDCl_3_): 176.03 (COMe), 154.11 (C-10),137.65 (C-2), 130.63 (C-13), 129.31 (C-8), 111.96 (C-11), 111–23 (C-12), 110.24 (C-9), 100.84 (C-7), 57.69 (C-21), 56.15 (OMe), 55.25 (C-16), 53.24 (C-5), 52.74 (CO2CH3), 51.60 (C-3), 39.27 (C-20), 36.67 (C-17), 32.14 (C-15), 27.44 (C-14), 26.86 (C-19), 22.33 (C-6), 11.82 (C-18). This compound was identified as voacangine by comparing its spectral and physicochemical data with those reported in the literature (Fig. 1c). Furthermore, compound 26SK002 exhibited the following physical and spectral properties: crystalline needles; MP:112–214 °C; ^**1**^ **H NMR** (300 MHz,CDCl_3_): 4.68 (1H, *d*, *J =* Hz, H-29ß), 4.57 (1H, *m,*H-29a), 4.49 (1H, *m,* H-3), 2.38 (1H, *m*, H-19), 2.04 (3H, *s*, H-2’), 1.90 (2H, *m*, H-21), 1.68 (3H, *s*, H30), 1.65 (2H, *m*, H-15), 1.61(2H, *m*, H-12), 1.51 (2H, *m*, H-6), 1.46 (1H, *m*, H-16), 1.38 (2H, *m*, H-18), 1.38 (2H, *d*, *J = 1.5* Hz, H-7), 1.27 (1H, *s*, H-9), 1.02 (3H, *s*, H-26), 0.93 (3H, *s*, H-27), 0.85 (3H, *s,* H-25), 0.84 (3H, *s*, H23), 0.83 (3H, *s*, H-24), 0.78 (3H, *s*, H-28). 23), 0.83 (3H, *s*, H-24) 0.78 (3H, *s*, H-28). ^**13**^ **C NMR** (75 MHz, CDCl_3_): 171.21 (C1’),151.15 (C-20), 109.50 (C-29), 81.10(C-3),55.49 (C-9), 48.39 (C-18), 48.14 (C-19), 43.13 (C-17), 42.94 (C-14), 40.96 (C-8), 40.13 (C-22), 38.49 (C-1), 38.14 (C13), 37.92 (C-10), 35.69 (C-16), 34.31 (C-7), 29.94 (C-21), 28.08 (C-2’), 27.55 (C23), 25.19 (C-12), 23.84 (C-12), 21.51 (C-2), 21.05 (C-11), 19.42 (C-30),18.33 (C6),18.14 (C-28), 16.64 (C-24), 16.33 (C-25), 16.10 (C-26), 14.63 (C-27). This compound was identified as lupeol acetate by comparing its spectral and physicochemical data with those reported in the literature (Fig. 1d).

### Determining the cytotoxic effects of the compounds

**Fig. 2:**
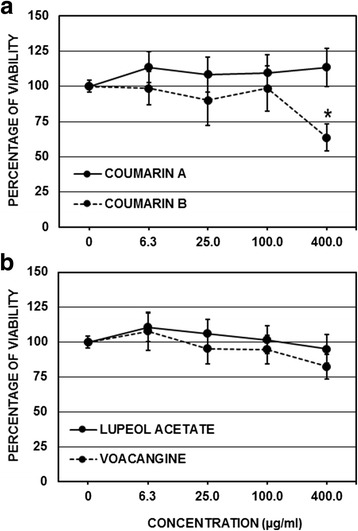
Evaluation of compound cytotoxicity in VERO cells. Each compound was evaluated by MTT at concentrations from 6.25 μg/mL to 400 μg/mL and compared with the untreated controls. **a** Compounds extracted from the seeds of *M. americana.* **b** Compounds extracted from the seeds of *T. cymosa*. *Only coumarin B significantly decreased the cellular viability at a concentration of 400 μg/mL (ANOVA-LSD, *p* < 0.05)

The CC_50_ of the coumarin A and B compounds, which were derived from the seeds of *M. americana*, were 3150.0 and 549.8 μg/mL, respectively. Furthermore, the CC_50_ of the lupeol acetate and voacangine compounds derived from *T. cymosa* were 4015.5 and 1136.3 μg/mL, respectively. Finally, by comparing the viability percentages of each of the compounds (with concentrations from 400 to 6.3 μg/mL) with the control without a compound, only coumarin B at 400 μg/mL significantly inhibited cellular viability, with a toxicity of 36.4% (*p* < 0.05, ANOVA-LSD) (Fig. 2). The cytotoxicity determination of the extracts was performed by MTT assay. The non-cytotoxic concentrations were used in the dose-response assays (Table 2).

**Table 2:** CC_50_, EC_50_, and SI values for the compounds derived from *M. americana* and *T. cymosa* in VERO cells infected with DENV-2/NG or CHIKV-ACol

		DENV-2/NG			CHIKV-ACol		
Scientific Name	Compounds	CC_50_ (μg/mL)	EC_50_ (μg/mL)	SI	CC_50_ (μg/mL)	EC_50_ (μg/mL)	SI
*Mammea americana*	Coumarin A 34SK001	3150.0	9.6	328.1	3150.0	10.7	295.2
	Coumarin B 34SK002	549.8	2.6	211.5	549.8	0.5	1021.0
*Tabernaemontana cymosa*	Lupeol Acetate 26SK001	4015.5	37.5	107.1	4015.5	538.5	7.5
	Voacangine 26SK002	1136.3	10.1	113.0	1136.3	304.3	3.7

### Determining the activity of the compounds on the viral infections

The EC_50_ of the compounds was determined after titrating the supernatants that had been obtained by assaying the inhibition of the production of infectious viral particles and at non-cytotoxic doses. In the VERO cell cultures that were infected with the DENV-2/NG strain and treated with the coumarin A or B compounds, the EC_50_ values were 9.6 and 2.6 μg/mL, respectively. In the cultures that were treated with the lupeol acetate or voacangine compounds, the EC_50_ values were 37.5 and 10.1 μg/mL, respectively. However, the SIs were above 100, with coumarin A (SI 328.1) being the most selective. In the VERO cell cultures infected with the CHIKV/ACOL strain and treated with the coumarin A or B compounds, the EC_50_ values were 10.7 and 0.5 μg/mL, respectively. In cultures that were treated with the lupeol acetate or voacangine compounds, the EC_50_ values were 538.5 and 304.3 μg/mL, respectively. Furthermore, only the coumarin A or coumarin B compounds were considered highly selective (SI 295.2 and 1021.0, respectively). The lupeol acetate and voacangine compounds were not considered selective because they had an SI value lower than10 (Table 2).

### Compound effects on the entry of the virus into the cell

**Fig. 3:**
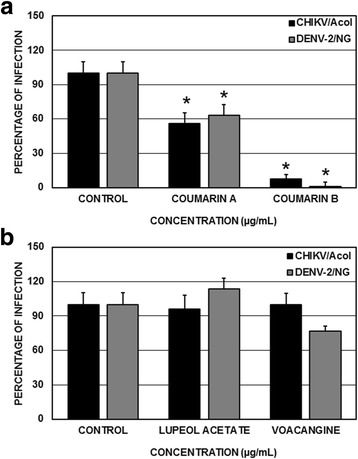
Antiviral effects on viral production (DENV-2/NG or CHIKV/ACol) using the pre-treatment strategy. The cells were treated with each compound at a concentration of 200 μg/mL and were subsequently infected. **a ** Effects of the compounds extracted from the *M. Americana* seeds. **b** Effects of the compounds extracted from the *T. cymosa* seeds*.* Statistically significant inhibitions are observed only in the cultures treated with coumarin A or coumarin B (Student’s t-test, *p* < 0.05)

To identify if any of the compounds was able to prevent viral entry into the cells, the compounds were added to the cell culture before viral infection was initiated (the pre-treatment strategy). Only the coumarin A and coumarin B compounds derived from the seeds of *M. americana* significantly inhibited CHIKV-ACol infection (with inhibition percentages of 44.0 and 92.5%, respectively) or DENV-2/NG infection (with inhibition percentages of 37.1 and 99.2.5%, respectively) (*p* < 0.05, Student’s *t*-test) (Fig. 3a). However, the inhibition percentages of the lupeol acetate and voacangine compounds, which were derived from the seeds of *T. cymosa*, were very low, both in cultures infected with CHIKV-ACol (at 4.1 and 0.4%, with respect to the untreated control) as well as those infected with DENV-2/NG (the inhibition did not surpass 23%) (*p* > 0.05, Student’s *t*-test) (Fig. 3b).

### Compound effects on viral replication

**Fig. 4:**
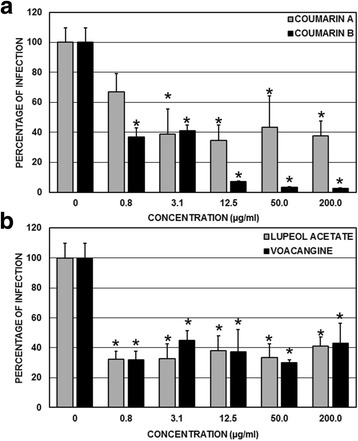
Antiviral effect on the viral genome replication of DENV-2/NG during the post-treatment strategy. The cells were infected and subsequently treated with the compounds at concentrations from 0.8 to 200 μg/mL and compared with the untreated controls. **a** Effects of the compounds extracted from the *M. Americana* seeds. **b** Effects of the compounds extracted from the *T. cymosa* seeds*.* Statistically significant inhibitions are observed in the cultures treated with coumarin A (concentrations greater than 3.1 μg/mL) or treated with coumarin B, lupeol acetate, or voacangine (all of the concentrations evaluated here) (ANOVA-LSD, *p* < 0.05)

The effect of the compounds on the steps subsequent to viral entry into the cells was evaluated after infection had begun. The compounds derived from the *M. americana* seeds significantly inhibited infection by DENV-2/NG. The coumarin A compound significantly inhibited infection by this virus at concentrations greater than 3.1 μg/mL (at inhibition percentages from 56.6% with respect to the untreated control) (*p* < 0.05, ANOVA-LSD). However, all the concentrations of the coumarin B compound (from 0.8 to 200 μg/mL) significantly inhibited infection in relation to the untreated control (*p* < 0.05, ANOVA-LSD), with percentages of inhibition greater than 58.8% and close to 100% (Fig. 4a). Finally, the two compounds derived from the *T. cymosa* seeds also significantly inhibited infection by DENV-2/NG. In the case of the lupeol acetate compound, all the compound concentrations (from 0.8 to 200 μg/mL) significantly inhibited infection in relation to the untreated control (*p* < 0.05, ANOVA-LSD), with inhibition percentages between 59.0 and 67.7%. This behavior was similar to that observed in cultures that were treated with the voacangine compound, in which all the concentrations significantly inhibited infection by inhibition percentages from 55.2 to 70.1%. (*p* < 0.05, ANOVA-LSD) (Fig. 4b).

**Fig. 5:**
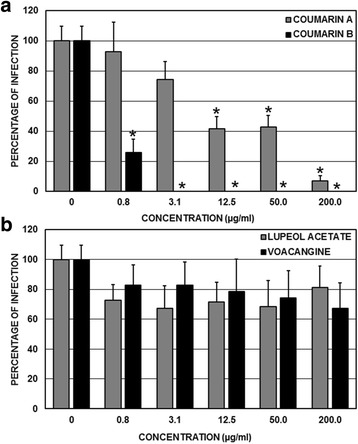
Antiviral effect on the production of infectious viral CHIKV/ACol particles during the post-treatment strategy. The cells were infected and subsequently treated with compounds at concentrations from 0.8 to 200 μg/mL and compared with the untreated controls. **a** Effect of the compounds extracted from the *M Americana* seeds. **b** Effect of the compounds extracted from the *T. cymosa* seeds. Statistically significant inhibitions are observed in the cultures treated with coumarin A (concentrations greater than 12.5 μg/mL) or coumarin B (all of the evaluated concentrations) (ANOVA-LSD, *p* < 0.05)

The compounds derived from the *M. americana* seeds also significantly inhibited infection by CHIKV-ACol. The coumarin A compound significantly inhibited infection by CHIKV-ACol at concentrations of 12.5, 50, and 200 μg/mL (at inhibition percentages of 58.6, 57.1, and 92.9%, respectively, with respect to the untreated control) (*p* < 0.05, ANOVA-LSD). Furthermore, all the concentrations of the coumarin B compound (from 0.8 to 200 μg/mL) significantly inhibited infection in relation to the untreated control (*p* < 0.05, ANOVA-LSD), with inhibition percentages of 100%, except for the 0.8 μg/mL concentration, which only inhibited 74.3% (Fig. 5a). Finally, the compounds derived from the *T. cymosa* seeds did not significantly inhibit infection by CHIKV-ACol. In the case of the lupeol acetate compound, no percentage of inhibition surpassed 33%, and in no cases were there statistically significant differences in relation to the untreated control (*p* > 0.05, ANOVA-LSD). The voacangine compound showed the same behavior, in which all the inhibition percentages were less than 33% without statistically significant differences in relation to the untreated control (*p* > 0.05, ANOVA-LSD) (Fig. 5b).

## Discussion

Despite the global distribution of DENV and CHIKV, the management of patients affected by the diseases that these viruses cause is still performed during an exclusively symptomatic form because of the absence of a specific antiviral treatment. In this context, our working group has focused its efforts on evaluating potential antivirals for several years, whether licensed medications [33, 35] or compounds derived from natural products. In the natural products area, we found that extracts derived from plants in the Colombian Caribbean region are capable of inhibiting DENV replication in vitro [26]. For this reason, an in vitro evaluation was conducted on compounds derived from the extracts of the seeds from two plants that were studied previously, namely *M. americana* and *T. cymosa*, which have been shown to have an antiviral effect against the DENV.

In this study, the ethanolic extract of *M. americana* seeds (FD-I-34S) was fractionated by open column chromatography, and the coumarinic type compounds 34SK001 and 34SK002 were characterized from the 34SF03 fraction (Table 1); the structures of these compounds were consistent with those reported in the literature, and therefore, we named them coumarin A and coumarin B, respectively (Fig. 1a-b) [36]. Subsequent to this characterization, the cytotoxicity of these compounds was evaluated, and it was found that the two coumarins are slightly toxic (causing a small reduction in cellular proliferation), with CC_50_ values of 3150.0 and 549.8 μg/mL, respectively (Table 2). Coumarin A was less toxic because coumarin B significantly inhibits the cellular viability in the cultures that had been treated with a concentration of 400 μg/mL (Fig. 2a). Although the antiproliferative activity of the coumarins has been described in tumoral cells [37], it is important to note that the VERO cells used in this study are not of the tumoral type, so the results are commensurate with the type of cell under evaluation. In addition, it is consistent with results that were recently reported by other authors, who found CC_50_ values higher than 75.2 μg/mL in this same type of cell [38].

When evaluating the effectiveness and selectivity of these two compounds on infection by DENV-2/NG, we found that the concentrations needed to inhibit 50% of viral replication are very low (9.6 μg/mL of coumarin A and 2.6 μg/mL of coumarin B). This result was consistent with that found in the assay of viral replication inhibition, in which it was shown that very low concentrations of either of the coumarins (3.1 μg/mL, for example) significantly inhibited the replication in relation to the untreated control (Fig. 4a). In the case of the cultures that were infected with CHIKV/ACol, although the concentrations that were necessary to inhibit 50% of viral production remained low (10.7 μg/mL for coumarin A and 0.5 μg/mL for coumarin B) (Table 2), only coumarin B significantly inhibited infection in comparison with the control at lower concentrations (0.8 μg/mL). Significant coumarin A inhibitions were presented at the 12.5 μg/mL concentration (Fig. 5a). Additionally, the largest SI found here was for coumarin B during infection by CHIKV/ACol (SI 1021.0).

In spite that was desiderable to compare the antiviral effect of extracts of compouns derivated of the same plants, there is not any report published about antiviral effect of extracts of Mammea and Tabernaemontana against CHIKV. However in the case of DENV, the antiviral effect found of the compounds is consisted with the antiviral of the extracts that we report previously.

Coumarins have been the object of investigation in recent years because of their biological activities as anticoagulants [39], anticarcinogenics [37], anti-inflammatories/antioxidants [40], and insecticides against the larvae and adults of arthropods such as *A. aegypti* and *Anopheles arabiensis* [41]. Their ability to inhibit the replication of various microorganisms, among which are *Leishmania amazonensis* [42], *Trypanosoma cruzi* [43], and *Mycobacterium tuberculosis* [44], and viruses, such as the human immunodeficiency virus type 1 (HIV-1) [45], and Hepatitis C [46], was also studied. In this context, our results are consistent with recent reports of the antiviral effect of certain coumarins on infection by CHIKV. In one of these studies, it was found that five coumarinic type compounds had SIs no higher than 11.5 [38]. A similar behavior has been reported for coumarins derived from the *Trigonostemon howii* plant, which also inhibited CHIKV replication, but only moderately (SI: 30) [47]. However, by using a CHIKV replicon cell line, a coumarin has been identified with an SI of 308 [48]. In our case, the SIs ranged from 295 to 1021, making our compounds better potential antivirals for this virus in relation to those that were previously reported. The anti-inflammatory activity of coumarins has been described [49], and considering that the CHIKV induces a disease that is also inflammatory [50], the possible effects of the coumarins in vivo could go beyond the antiviral effect. Subsequent studies in animal models should focus on both fronts.

This study would be the first report of the antiviral effect of coumarins on DENV-2/NG infection in a cell culture system. This antiviral effect could be due to the inactivation of viral particles by the coumarins because of the joining of the pyrimidines present in the viral nucleic acid, a mechanism used by psoralen (a compound derived from the coumarins) to inactivate DENV-1. This compound is used as an immunogen in vaccine trials [51] or to inhibit the activity of the NS5 viral protein (which actively participates in viral replication), a mechanism that has been demonstrated in coumarins derived from the *Myrtopsis corymbosa* plant by enzyme inhibition assays [52].

It is important to note that the coumarins (specifically those derived from the *Mammea neurophylla* plant) have been demonstrated to have beneficial effects on the endothelial dysfunction generated by diseases such as diabetes [53]. Taking into account that the severe forms of dengue are specifically associated with endothelial dysfunction [54], the future use of these coumarins as an effective treatment could prevent the development of severe forms of the disease. Additionally, it is important to note that it has also been reported that some coumarins inhibit the production of nitric oxide [55] and that, in turn, the nitric oxide exerts an innate antiviral role during DENV infection (decreasing the amounts of protein, genome, and infectious viral particles) [55]. Thus, further studies would be needed using in vivo models to check the actual beneficial effects on the pathogenesis of the infection.

In addition, the ethanolic extract of the *T. cymosa* (FD-I-26S) seeds was also fractionated by open column liquid chromatography, and the compounds 26SK001 and 26SK001 from fraction 26SF01 were characterized (Table 1). The structure of the first compound is consistent with that reported in the literature for voacangine [56], and that of the second is consistent with that reported for lupeol acetate [57] (Fig. 1c–d). Subsequent to the identification, their toxicity in VERO cells was evaluated, showing that neither of the two compounds at the highest concentrations used here (400 μg/mL) could inhibit cellular proliferation in comparison with the untreated control, which makes them much less toxic in this cell system (Fig. 2b). For the coumarins, it has been reported that voacangine inhibits the proliferation of endothelial tumor cells [58], and in non-tumor cells, a CC_50_ greater than 400 μg/mL has been reported, which is consistent with our results [59]. In the case of lupeol acetate, although there are no reports on its anti-proliferative activity, it has been demonstrated that the CC_50_ of lupeol (a structurally related compound) in VERO cells is greater than 300 μg/mL, which is also consistent with our results [60].

Unlike the antiviral effect that we report for the coumarins derived from *M. americana*, which are effective both for DENV-2/NG as well as for CHIKV/ACol, the compounds derived from *T. cymosa* (voacangine and lupeol acetate) are only effective against infection by DENV-2/NG (Figs. 4b and 5b), significantly inhibiting viral replication from the lowest evaluated concentrations (0.8 μg/mL). This result is consistent with the calculated SIs, which are greater than 100 for DENV-2/NG and less than 10 for CHIKV/ACol (Table 2).

Lupeol acetate forms part of a series of compounds known as pentacyclic triterpenes, among which lupeol is the most studied. The inhibitory effect of this family of compounds has been reported for different viruses, including Japanese encephalitis, tick-borne encephalitis, West Nile virus, and DENV [61]. However, very few reports are present in the literature that cover the biological activity of lupeol acetate specifically, but recently, its anti-inflammatory activity has been shown to decrease the synthesis of TNF-α and IL-2 and increase the synthesis of IL-10 [62]. Additionally, it also decreases the number of iNOS cells, suggesting an active role in the synthesis of pro-inflammatory cytokines and in the nitric oxide system [63]. Although the discovery of the anti-replicative effect of DENV is very interesting and has been reported here for the first time, the adverse effects that a possible treatment with this compound could have should be handled carefully. In mice with rheumatoid arthritis, there is an increase in IL-10 [64], and this cytokine is one of the primary factors that is responsible for the development of severe forms of dengue [61].

Voacangine belongs to the group of indole alkaloids that are commonly distributed among flowering plants [65]. There are few reports of its biological activity, but it has been found to have an antimicrobial action against gram-positive bacteria [52] and various species of *Mycobacteria* [66]. Thus far, there is no report about its action on viruses; therefore, our study is the first report of its antiviral activity. However, biocomputational techniques were recently used to show that several indole alkaloids have a high affinity for nonstructural proteins in DENV (such as NS2B-NS3, NS3, and NS5), which could explain the inhibitory effect of voacangine on viral replication, considering that these proteins are active in this process [67]. However, DENV-2 has been shown to increase the synthesis of vascular endothelial growth factor (VEGF), which is involved in the development of severe forms of the disease [68]. Furthermore, it has been demonstrated that voacangine inhibits the angiogenesis mediated by this factor, which could cause an in vivo infection. The beneficial effects of the compounds were beyond those produced by replication inhibition [58]. Further studies are needed to elucidate these possible mechanisms.

## Conclusions

Our results reported the antiviral activity of four compounds derived from plants in the Colombian Caribbean region against infection by two viruses (DENV and CHIKV) that are endemic in many tropical and subtropical regions, including Colombia. The coumarins were shown to be potent in vitro antivirals for both viruses (the inhibition percentage of the infection was close to 100%), while that of lupeol acetate and voacangine are effective only against DENV, demonstrating the differential effectiveness of these two compounds. Subsequent studies with in vivo models could help to elucidate the antiviral mechanism induced by these two compounds as well as other possible effects beyond that of the antivirals that can help improve the pathogenesis of dengue and chikungunya.

## Abbreviations

CC_50_: 50% cytotoxicity concentration
CHIKV: Chikungunya virus
DENV: Dengue virus
EC_50_: 50% effective concentration
PBS: Phosphate-Buffered Saline
SI: Selectivity Index
WHO: World Health Organization
